# Wearing a Mask Shapes Interpersonal Space during COVID-19 Pandemic

**DOI:** 10.3390/brainsci12050682

**Published:** 2022-05-23

**Authors:** Monica Biggio, Ambra Bisio, Valentina Bruno, Francesca Garbarini, Marco Bove

**Affiliations:** 1 Department of Experimental Medicine, Section of Human Physiology and Centro Polifunzionale di Scienze Motorie, University of Genoa, Viale Benedetto XV 3, 16132 Genova, Italy; monica.biggio@edu.unige.it (M.B.); ambra.bisio@unige.it (A.B.); 2 MANIBUS Lab, Department of Psychology, University of Turin, Via Verdi 10, 10124 Turin, Italy; valentina.bruno@unito.it (V.B.); francesca.garbarini@unito.it (F.G.); 3 IRCCS Ospedale Policlinico San Martino, Largo Rosanna Benzi 10, 16132 Genova, Italy

**Keywords:** SARS-CoV-2, social distancing, protective aid, peripersonal space, interpersonal space, reaching space, comfort space

## Abstract

Social distancing norms have been promoted after the COVID-19 pandemic. In this work, we tested interpersonal space (IPS) in 107 subjects through a reaching-comfort distance estimation task. In the main experiment, subjects had to estimate the comfort and reach space between an avatar wearing or not wearing a face mask. We found that IPS was greater between avatars not wearing a mask with respect to stimuli with the mask on, while reaching space was not modulated. IPS increment in the NoMask condition with respect to the Mask condition correlated with anxiety traits, as shown with the State-Trait Anxiety Inventory, rather than with transient aspects related to the pandemic situation. In the control experiment, the avatars with a mask were removed to further explore the conditioning effect provided by the presence of the facial protection in the main experiment. We found a significant difference comparing this condition with the same condition of the main experiment, namely, the distances kept between avatars not wearing a mask in the main experiment were greater than those between the same stimuli in the control experiment. This showed a contextual adaptation of IPS when elements related to the actual pandemic situation were relevant. Additionally, no significant differences were found between the control experiment and the Mask condition of the main experiment, suggesting that participants had internalized social distancing norms and wearing a mask has become the new normal. Our results highlight the tendency of people in underestimating the risk of contagion when in the presence of someone wearing a mask.

## 1. Introduction

Coronavirus disease 2019 (COVID-19) has rapidly changed our habits, also due to social guidelines promoted by various countries in order to limit the virus spreading. Consequently, social distancing is a concept that has entered daily language and has become familiar. Various governments have imposed “stay-at-home” policies, encouraging people to adopt “personal distancing” behaviors to reduce virus transmission. For instance, avoiding physical contact or close proximity with non-household members, reducing the use of shared public spaces, and maintaining at least 1 m of distance between yourself and others [1]. Furthermore, wearing of face masks has been imposed to protect people against infection [2], and even if other restrictions have been removed a year later, wearing a mask in public is still required. As in a Shakespeare’s comedy, we shall everyone be mask’d!

Although effective and essential for containing the virus, these social interventions imposing social distancing and isolation may greatly affect mental health [3,4], as observed following earlier epidemics, such as the 2003 SARS outbreak [1,5,6,7]. For example, boredom, separation from loved ones, financial problems, and uncertainty over the situation was shown to significant psychological distress, depressive symptoms, and also to forms of post-traumatic stress [6].

In normal conditions, the distance we choose to maintain between ourselves and others is a behavioral indicator of how close we prefer to stand relative to another person. The distance between two people in social interaction is defined as interpersonal space (IPS), and is critical in determining a successful social interaction and reducing the feelings of discomfort due to IPS violations [8,9,10,11,12,13,14,15].

The boundaries of IPS are not fixed, and are dynamically modulated by culturally rooted high-level factors, such as sex, age, and familiarity and intimacy with the other person [10,11,13,16,17,18,19,20]. In addition, evaluating the preferred interpersonal distance with virtual protagonists (friend or stranger), Perry and colleagues showed that individuals with social anxiety were characterized by attenuated early electrophysiological responses (especially the N1 component of the evoked potential), suggesting discomfort at an earlier stage than others in social engagement, which may lead them to stand further away [21].

In this time of forced isolation, it may be that the imposed social norms have influenced how we perceive others, who represent both a possible threat to our health and also the solution to our need for social interaction. In this regard, the stay-at-home and social distancing orders of the COVID-19 pandemic have highlighted the dynamics of proxemic behaviors [22]. Recently, the pandemic situation has led to a proliferation of articles exploring IPS in various contexts. Some of those focused on the role of the mask [23] and contagiousness [24] in the perception of others, while some others concentrated on the explicit temporal component of the IPS changes before and after the pandemic [25]. Here, we aimed to explore how pandemic containment policies, namely social distancing and mask wearing, changed IPS, investigating the extension of comfortable space between people during the lockdown period in Italy (June–November 2020). To ensure the safety of the experimental procedure, we employed an online version of the stop-distance task [8,19] using an analog scale resembling the interpersonal visual analog scale [18] and the Pedersen personal space scale [26], evaluating both the reaching and the comfort distance of subjects. This procedure is a frequently used paradigm for assessing preferred or tolerated IPS (comfort distance) in comparison to a more stable reaching distance, usually the length of one’s arm [19].

Through this technique, our work aimed to achieve two main goals: to study IPS changes by comparing the effect of seeing others wearing a mask (main experiment), and to explore whether social distancing norms have been internalized by people and have led to IPS changes when compared to a neutral situation (main vs. control).

## 2. Materials and Methods

### 2.1. Participants

Participants voluntarily took part in an online video version of the reaching-comfort distance estimation task [19] in a period between June and November 2020. The protocol was approved by the Ethical Committee of the University of Turin (prot. no. 251260), and both experiments were preceded by informed consent and the description of the task. A total of 107 subjects (age ± SD: 32.24 ± 11.89 years, 55 women) took part in two experiments (see Table 1). All participants were Italians living in Italy.

According to the questionnaire administered to subjects, the number of participants personally affected by COVID-19 was 16 for the main experiment and 5 in the control group, which was 19.51% and 20% of the total, respectively, comparable with the percentage of positive testing in that period.

### 2.2. Experimental Setup

**Figure 1:**
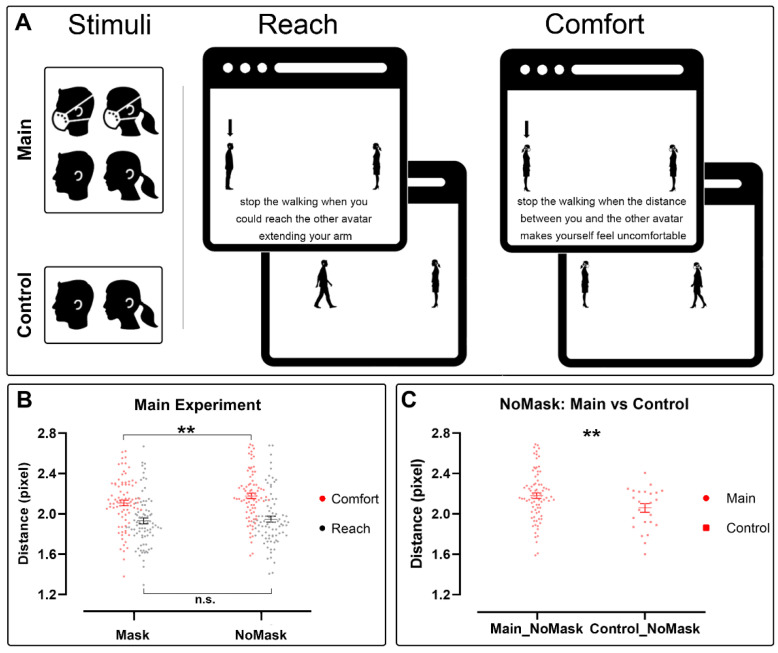
(**A**) Experimental design. Online version of reach-comfort distance estimation task. Subjects were asked to identify with the avatar (both male or female avatar, depending on the sex of the participant) indicated by the arrow on the first web page and follow the instruction written below. On the following page, one of the two avatars moved toward the other. Participants were asked to press the spacebar to stop the video when the moving avatar reached the target distance, either the comfort or the reach distance depending on the previous instruction. In the main experiment, avatars wearing or not wearing a mask were balanced, while in the control experiment, stimuli with the mask were removed. (**B**) Main experiment results. Distances between avatars in the Mask and NoMask conditions, and in comfort (red) and reach (grey) tasks. Distances were computed as the logarithmic value of the pixel between one avatar and the other. The continuous lines and the associated error bars indicate average values ± ES. ** refers to *p* < 0.01. (**C**) Comparison between main and control experiment. Change in the distance in the NoMask condition of the two experiments. Dots represent averaged data ± ES of NoMask condition in main experiment. Squares represent the NoMask condition in control experiment. Distances values are represented by the logarithmic value of the pixel between one avatar and the other. ** refers to *p* < 0.01.

A reaching-comfort distance estimation task was performed for both main and control experiments. To handle the display of visual stimuli and the keyboard response collection, we used a jsPsych procedure [27]. The whole experimental procedure took about 20 min. Subjects had to watch videos displayed online showing two avatars (black human silhouettes over a white background) walking toward each other in a third-person perspective. (Figure 1A). Following the instruction, subjects had to press the spacebar of their PC when the avatars reached the comfort distance or the reaching distance.

In the main experiment, half of the avatars wore protective aids (masks), while in the control experiment, only stimuli without masks were present. 

Additionally, a questionnaire regarding the subject’s description and information about personal contact with COVID-19 was displayed, as well as the STAI. In the main experiment, the questionnaire was displayed before the actual reaching-comfort distance estimation task. In the control experiment, in order to avoid subject conditioning, the questionnaire as well as an explanation of the aim of the study was administered after the actual task. 

#### 2.2.1. Main Experiment

Eighty-two participants (age ± SD: 32.72 ± 11.87 years, 44 women) underwent the experiment, consisting of watching videos showing two avatars walking toward each other. Before each trial, one of the two avatars (same sex of the participant) was pointed by an arrow. The participant was asked to identify themself with the indicated avatar. Then, one of the two avatars approached the other. Subjects were asked to press the spacebar to stop the video when the moving avatar reached the target distance, namely either the comfort or the reach distance. In half of the video, the comfort distance was estimated. Trials were preceded by the instruction: “stop the movement as soon as the distance between you and the other avatar makes yourself feel uncomfortable”. The other half of the trials referred to the reaching distance and were preceded by the following instruction: “stop the movement when you could reach the other avatar extending your arm”. Before the main task, participants were asked to fill out a form with some descriptive and personal information, such as the height, the region of origin, the region where they spent the lockdown period, the age, and particularly, if they had been tested for COVID-19. After that, subjects underwent an Italian version of the State-Trait Anxiety Inventory [28]. STAI is a self-report 40-item scale divided into two subscales that evaluate the current state of anxiety (State Anxiety Scale—S-Anxiety or STAI - S) and the general anxiety proneness (Trait Anxiety Scale—T-Anxiety or STAI - T). Responses for the S-Anxiety scale assess the intensity of current feelings “at this moment”: (1) not at all, (2) somewhat, (3) moderately so, and (4) very much so. Responses for the T-Anxiety scale assess the frequency of feelings “in general”: (1) almost never, (2) sometimes, (3) often, and (4) almost always. The reversed score items, indicating absence of anxiety, include: items 1, 2, 5, 8, 10, 11, 15, 16, 19, and 20 (State Anxiety Scale) and items 21, 23, 26, 27, 30, 33, 34, 36, and 39 (Trait Anxiety Scale). The items are then summed to obtain scores for state anxiety and for trait anxiety (items indicating absence of anxiety are reversed scored), with a range of possible scores from 20 to 80 for each measure of anxiety.

#### 2.2.2. Control Experiment

To understand whether subjects were unconsciously conditioned by the contextual information provided in the main experiment (i.e., mask stimuli, questions about COVID), a new group of 25 subjects (age ± SD: 30.68 ± 12.08 years, 11 women) underwent the control experiment. They were first displayed the block of videos. The control experiment had the same conditions as the main experiment, except for the videos showing avatars wearing a mask, which were removed. The STAI and the personal questions were asked at the end of the stop-distance estimation paradigm, and no mention or reference to COVID-19 was made before this moment. After that, participants were asked to fill out the same form as in the main experiment with some descriptive information.

### 2.3. Statistical Analysis

The outcome of interest of the experiment was the distance left between the avatars at the moment of the spacebar press. 

When the participants pressed the spacebar, the computer recorded the time interval between the beginning of each video and the subject’s response. The starting distance in pixel between the two avatars was known and fixed. For each trial, the target distance between the two avatars was computed as follows:

(1) $$Target\;distance=Starting\;Distance-\frac{Starting\;Distance\;*\;Response\;Time}{Total\;Video\;Duration}$$

We considered as dependent factors eight total conditions for the main experiment, on the basis of two tasks (comfort and reach), the mask (Mask and NoMask) and the movement of the avatar indicated by the arrow (walking and still), and four conditions for the control experiment.

The response distribution was checked with the Shapiro–Wilk test, and due to lack of normality, a logarithmic transformation was applied over all data.

Before running the analysis on dependent variables, we performed two multiple regression analyses, separately for the main and the control experiment, to verify that characteristics of the groups, namely the sex, the height, and being affected by COVID, were not significant predictors for the distance data.

In order to evaluate whether the distance was differently modulated by an avatar wearing or not wearing a mask or moving toward or waiting for the approach of the participants’ avatar, averaged distances recorded in the main experiment were analyzed by means of ANOVA with TASK (comfort vs. reach), MASK (Mask vs. NoMask), and MOTION (walking vs. still) as within factors.

Data collected in the control experiment were analyzed by means of ANOVA with TASK (comfort vs. reach) and MOTION (walking vs. still) as within factors.

Newman–Keuls post hoc analysis was used to interpret significant interactions. Values are presented as mean ± standard errors.

Further, in order to discern whether it was the distance while wearing a mask that decreased or the distance without the mask that increased, we compared the comfort distance of the main experiment in both the Mask and NoMask condition with the comfort distance of the control experiment data. Due to unbalanced sample sizes, we performed two separate Welch’s *t*-test for independent samples [29], comparing control vs. Mask and control vs. NoMask (all conditions averaged).

STAI questionnaire reliability coefficients were computed in Cronbach alpha, showing an excellent reliability of α = 0.93.

To evaluate whether the anxiety could be predictive of the increase in the comfort distance, the result of the STAI questionnaire was correlated with the difference between the NoMask and Mask distance in the comfort task. In particular, Spearman’s rank correlation coefficient was performed between the (NoMask vs. Mask) distance and STAI-T averaged values, STAI-S averaged values, STAI-T single items, and STAI-S single items. Significant correlations were considered after Bonferroni correction for multiple comparisons (0.05/20 items, *p* = 0.0025).

## 3. Results

### 3.1. Main Experiment

Multiple regression that was run for the main experiment showed that sex, height, and COVID-19 were never predictors for distances (see Table 2).

The following analysis of variance for distance in the main experiment revealed significant main effects for TASK (comfort vs. reach: 2.14 ± 0.01 pixel vs. 1.94 ± 0.02 pixel, respectively, F(1,81) = 63.28, *p* < 0.001) and MASK (Mask vs. NoMask: 2.02 ± 0.01 pixel vs. 2.07 ± 0.02 pixel, respectively, F(1,81) = 10.38, *p* < 0.01), and a significant interaction for TASK * MASK factors (F(1,81) = 6.55, *p* = 0.012). Post hoc analysis of the interaction revealed a significant decrease in the distance in the Mask condition with respect to the NoMask condition in the comfort task (comfort Mask vs. comfort NoMask: 2.11 ± 0.02 pixel vs. 2.18 ± 0.02 pixel, respectively, *p* < 0.001), but not in the reach task (reach Mask vs. reach NoMask: 1.93 ± 0.02 pixel vs. 1.95 ± 0.02 pixel, respectively, *p* = 0.246). Furthermore, distances in comfort Mask and comfort NoMask conditions were always significantly larger than reach Mask and reach NoMask conditions (*p* always > 0.001). Results are presented in Figure 1B.

### 3.2. Control Experiment 

Multiple regression that was run for the control experiment showed that sex, height, and COVID-19 were never predictors for distances (see Table 2). 

ANOVA for distance in the control experiment revealed significant main effects for TASK, indicating that distance was significantly greater in the comfort (2.06 ± 0.04) with respect to the reach (1.90 ± 0.04) task (F(1,24) = 16.51, *p* < 0.01), as observed in the main experiment.

### 3.3. Comparison between Distance in Main Experiment and Control Experiment

A *t*-test comparing the comfort distance in the control experiment and in the NoMask condition of the main experiment revealed a significant difference, showing that the distance in the NoMask condition was significantly larger than in the control experiment (control vs. NoMask: 2.06 ± 0.04 vs. 2.18 ± 0.02, respectively, t(105) = −2.71, *p* < 0.01). This result is presented in Figure 1C and summarized in Table 3. Conversely, the analysis comparing the control experiment and the Mask condition of the main experiment showed no significant effects (control vs. Mask: 2.06 ± 0.04 vs. 2.11 ± 0.003, respectively, t(105) = −1.41, *p* = 0.16).

### 3.4. STAI Correlation

Results of the Spearman’s rank correlation indicated no correlation between the difference between the NoMask and Mask conditions in the main experiment’s comfort task and items of the STAI—S scale. Conversely, the analysis revealed significant positive correlations with the STAI—T items: “I get in a state of tension or turmoil as I think over my recent concerns and interests” (rho(82) = 0.38, *p* = 0.0018) and a tendency toward significance with the item: “I feel like a failure” (rho(82) = 0.30, *p* = 0.0026). Correlations and STAI scores are summarized in Table 4.

## 4. Discussion

During the Italian lockdown period, between June and November 2020, we reached 107 participants via online advertisements of the University of Genoa and Turin. Volunteers underwent an online version of the reaching-comfort distance estimation task. Subjects had to observe avatars moving and stop their walking at the minimum reach or comfort distance. 

In the main experiment, 82 subjects watched couples of avatars of both sexes wearing a mask, alternating with couples with bare faces. Twenty-five subjects underwent the control experiment, in which stimuli of avatars wearing masks were removed in order to understand whether participants were unconsciously conditioned by the contextual information provided in the main experiment, where mask and no mask stimuli were alternated. 

In the main experiment, we asked subjects to stop approaching avatars, wearing or not wearing a mask, at a comfort and reaching distance, showing that subjects maintained a greater comfort distance when avatars did not wear facial protection. Results showed that only the comfort space was significantly modulated by the presence or absence of the mask. In particular, subjects maintained a significantly greater comfort distance between avatars not wearing masks with respect to avatars wearing masks. In contrast, the reaching distance (which was always smaller than the comfort distance) was not modulated by the presence or absence of the mask. Reaching distance is often associated with the active peripersonal space (PPS) [30], a sensorimotor representation of the space in which we can directly interact with the surrounding world [31,32,33,34], in contrast with its defensive function where its role is protecting the body from dangerous stimuli surrounding it [35]. The active function of PPS and the social space coded by IPS are related, but has shown to be dissociable [13,36]. This result suggests greater stability of the active space in the social context [13], and an influence of the mask over the social dimension only. More interestingly, it showed that in the pandemic period, when social distancing policies were stressed, the “mask” factor constituted an element of awareness, and the proxemic behavior was subsequently modulated. This is in line with the recent literature exploring IPS and COVID-19. For example, since the presence of a face mask could impair emotional identification [37], Cartaud and colleagues investigated how faces wearing a mask are perceived with respect to those with a neutral, angry, or happy expression. They found that masked figures were perceived as more trustworthy than others, leading subjects to indicate shorter preferred distances [23].

The effect of contracting COVID-19 was evaluated as a predictor of both the reach and comfort distance, considering separately the group of subjects that had been affected by COVID-19 in comparison to the rest of the participants. We hypothesized that subjects who directly experienced COVID-19 disease would be more aware of the proximity between people, potentially showing an increased comfort distance, or conversely, an illusory feeling of security given by the immunity to the virus. On the contrary, our model showed that being in contact with the virus did not modulates subjects’ responses. The boundaries of interpersonal space are not fixed, and our results showed that contextual rules, such as social distancing norms, could be learned and applied in the correct situation, modifying IPS extension when necessary. This means that these modifications are not stable, but can be learned and applied when the situation demands them, as shown by the differences we found between the main and the control experiments. 

The intensity of this modification was, however, modulated by invariable characteristics of subjects. This was suggested by the absence of the influence of suffering from COVID-19, showing that previous experiences did not influence IPS modulation, in contrast with the fact that a general proneness to deal with recent concerns with anxiety was related to behavioral results. This was also supported by the correlation between personality traits related to anxiety and IPS enlargement in the absence of a mask, as reported afterward. Subjects filled out the Spielberger State-Trait Anxiety Inventory (STAI) [28], a scale that evaluates both the current state of anxiety and general anxiety proneness. The difference between the NoMask and Mask conditions in the comfort task of the main experiment showed a significant positive correlation with the trait items of STAI: “I get in a state of tension or turmoil as I think over my recent concerns and interests”. This item seems related to the subject’s general proneness to experience anxiety when thinking about their preoccupations (trait), with respect to a specific anxious response to the actual situation (state). This shows that IPS modifications did not depend on the delicate circumstances during the pandemic since no correlations were found with items investigating the state of anxiety, but they were otherwise related to anxious personality traits [38]. IPS modulation seems to be more related to stable aspects of personality than with transient aspects of anxiety related to the pandemic situation.

Analysis of the control experiment, in which the mask factor was absent, revealed a significant difference between the tasks, indicating that distance was significantly greater in the comfort with respect to the reach task as observed in the main experiment. This result replicated the literature data, in which comfort distance is greater than the reach space when in the presence of a stranger [17].

The comparison between the main and control experiments suggested an enlargement of interpersonal space between avatars without a mask, and not a reduction in distances between figures with facial protection on. Stimuli in the control experiment were the same as in the Main_No Mask condition. The only differences were the order of the exposition to the instructions, and that in the main experiment, stimuli showing avatars without a mask were alternated with stimuli wearing masks, making the social situation that subjects were living during the lockdown period relevant, during which our experiments took place [39]. In particular, the comparison showed that the comfort space indicated by subjects in the Main_No Mask condition was significantly larger than that maintained in the control experiment. Differently, the distance in the Main_Mask condition and that in the control experiment were comparable. It is possible that, unconsciously or not, subjects considered individuals not wearing a mask as a greater threat to their own safety. The significant difference between the Main_No Mask condition and control experiment in terms of the comfort distance, together with the lack of differences between the latter and the Main_Mask condition, indicates that the condition in which avatars did not wear a mask was perceived differently in the main and control experiments, even if the stimuli were the same. In this view, the interpersonal distance in the NoMask condition increased, while the Mask condition became the new normal. This is in line with previous studies that have associated loneliness and reduced social contact, both self-reported [40] and experimentally modulated by keeping people alone for at least 45 min before testing IPS [41]. 

We acknowledge that a limitation in our work is the small sample of the control group; however, our results are in line with Welsch and colleagues [25]. They modulated the time and asked people to imagine themselves before, during, and after the pandemic and tested the IPS with an avatar without considering the factor of the mask. They found that during pandemic, people had larger IPS than before or after. They asked explicitly to focus on the time, while we tested this implicitly. 

Further improvements of our work should include a mixed condition where only one of the two avatars wears a mask, either the one representing the subject or the “other”. Those conditions may allow one to explore the risk assessment of being protected by a mask when someone approaching is not wearing a mask, or the perceived threat when the subject is not covered by a mask. 

To summarize, in the present work, we pointed out that wearing a mask shaped the interpersonal space. Our study highlights that mask use modulated subjects’ perception of their comfort space. However, this was true specifically in the case of the avatars not wearing any facial protection. There may be various explanations for this phenomenon: for example, subjects could have internalized social distancing norms, particularly when wearing masks, but also, masks may lead to overconfidence in subjects, biasing their risk assessment. However, our results suggest a potentially harmful situation since subjects felt probably safer when wearing and in the presence of someone with a mask, and they may underestimate the risk of proximity with people wearing facial protection. This may constitute an important message for political and social interventions requiring social distancing, which also need to promote the importance of being apart even while wearing masks [42] and are not an alternative to these measures. Additionally, since people tend to underestimate the risk when in the presence of someone wearing protective aid, it is necessary to stress the importance of using filtering masks giving the correct protection, not reused nor cloth or aesthetic masks [43]. 

To conclude, IPS representation during pandemic is changing. People without facial protection are considered a possible threat for the health and masks are becoming a symbol of protection, as well as our new normal. Our work highlights the delicate balance between the need for socialization and the fear of meeting someone without hiding behind a mask.

## Figures and Tables

**Table 1:** Descriptive of the main and control experimental group.

Characteristics	Number	Percentage %	Characteristics	Number	Percentage %
Main Experiment			Control Experiment		
Age, years (±SD)	32.72 ± 11.87		Age, years (±SD)	30.68 ± 12.08	
Height, cm (±SD)	171.51 ± 8.70		Height, cm (±SD)	169.92 ± 8.93	
Sex			Sex		
Women	44	53.66%	Women	11	44%
Men	38	46.34%	Men	14	56%
COVID			COVID		
No	66	80.49%	No	18	72%
Yes	16	19.51%	Yes	7	28%

**Table 2:** Multiple regression with sex, height, and COVID-19 as predictors of subject responses.

**Main Experiment**			
	**R^2^**	**F (3–78)**	***p***
Comfort—Mask—stop	0.018	0.466	0.707
Comfort—Mask—go	0.016	0.414	0.744
Comfort—NoMask—stop	0.015	0.393	0.759
Comfort—NoMask -go	0.051	1.411	0.246
Reach—Mask—stop	0.092	2.619	0.157
Reach—Mask—go	0.050	1.373	0.257
Reach—NoMask—stop	0.080	2.275	0.186
Reach—NoMask—go	0.041	1.109	0.351
**Control Experiment**			
	**R^2^**	**F (3–21)**	***p***
Comfort—stop	0.043	0.312	0.817
Comfort—go	0.054	0.398	0.756
Reach—stop	0.068	0.507	0.682
Reach—Go	0.181	1.544	0.233

**Table 3:** ANOVAs comparing comfort and reaching distance in the different conditions. Upper: main experiment; Lower: control experiment.

**Main Experiment**			
	**df**	**F**	***p***
TASK (comfort vs. reach)	1, 81	63.28	0.000 **
MASK (yes vs. no)	1, 81	10.38	0.002 **
MOTION (walking vs. still)	1, 81	1.03	0.312
TASK * MASK	1, 81	6.55	0.012 *
TASK * MOTION	1, 81	2.57	0.113
MASK * MOTION	1, 81	1.61	0.208
TASK * MASK * MOTION	1, 81	0.59	0.445
**Control Experiment**			
	**df**	**F**	***p***
TASK (comfort vs. reach)	1, 24	16.507	0.000 **
MOTION (walking vs. still)	1, 24	0.037	0.848
TASK * MOTION	1, 24	0.628	0.436

* significant effect for *p* < 0.05. ** significant effect for *p* < 0.01.

**Table 4:** Spearman’s correlation between STAI items and the difference between NoMask and Mask conditions in the main experiment. Multiple comparisons’ significant threshold levels were adjusted with Bonferroni correction (0.05/20 items, *p* = 0.0025).

STATE ITEM	Main (M ± ES)	rho(82)	*p*	TRAIT ITEM	Main (M ± ES)	rho(82)	*p*
S-STAI1−	2.04 ± 0.07	0.149	0.182	T-STAI1−	1.63 ± 0.08	−0.049	0.659
S-STAI2−	2.05 ± 0.07	0.095	0.398	T-STAI2+	2.05 ± 0.08	0.162	0.147
S-STAI3+	1.88 ± 0.07	0.095	0.398	T-STAI3−	2.20 ± 0.09	0.080	0.473
S-STAI4+	1.96 ± 0.07	−0.132	0.238	T-STAI4+	1.85 ± 0.09	0.282	0.010
S-STAI5−	2.15 ± 0.07	−0.024	0.832	T-STAI5+	1.43 ± 0.07	0.302	0.006
S-STAI6+	1.62 ± 0.07	0.098	0.381	T-STAI6−	2.66 ± 0.09	−0.114	0.307
S-STAI7+	1.68 ± 0.07	0.155	0.165	T-STAI7−	2.27 ± 0.08	0.028	0.801
S-STAI8−	2.23 ± 0.07	0.071	0.525	T-STAI8+	1.78 ± 0.07	0.037	0.744
S-STAI9+	1.39 ± 1.07	0.241	0.029	T-STAI9+	2.17 ± 0.10	0.173	0.119
S-STAI10−	2.01 ± 1.07	0.094	0.401	T-STAI10−	1.84 ± 0.10	0.162	0.147
S-STAI11−	2.16 ± 0.07	0.078	0.486	T-STAI11+	1.96 ± 0.08	0.048	0.672
S-STAI12+	1.80 ± 0.07	0.150	0.179	T-STAI12+	1.89 ± 0.09	−0.002	0.988
S-STAI13+	1.74 ± 1.07	0.191	0.086	T-STAI13−	1.87 ± 0.11	0.080	0.475
S-STAI14+	1.90 ± 1.07	0.133	0.233	T-STAI14−	2.32 ± 0.13	0.081	0.469
S-STAI15−	2.33 ± 0.07	0.084	0.455	T-STAI15+	1.76 ± 0.08	0.110	0.324
S-STAI16−	2.20 ± 0.07	0.209	0.059	T-STAI16−	1.60 ± 0.09	0.083	0.459
S-STAI17+	1.94 ± 0.07	0.140	0.208	T-STAI17+	2.01 ± 0.09	−0.019	0.868
S-STAI18+	1.60 ± 0.07	0.023	0.835	T-STAI18+	2.07 ± 0.09	0.136	0.223
S-STAI19−	2.63 ± 1.07	−0.003	0.976	T-STAI19−	1.73 ± 0.10	−0.054	0.633
S-STAI20−	1.99 ± 0.07	0.070	0.535	T-STAI20+	2.54 ± 0.09	0.375	*0.001 ^1^*
**SUM state**	**39.30 ± 9.07**	0.101	0.367	**SUM trait**	**39.62 ± 0.95**	0.171	0.125

^1^ significant effect for *p* < 0.0025 after Bonferroni correction for repeated measures.

## Data Availability

The datasets used and/or analyzed during the current study are available from the corresponding author on reasonable request.
